# Trends in Encounters for Emergency Contraception in US Emergency Departments, 2006-2020

**DOI:** 10.1001/jamanetworkopen.2023.53672

**Published:** 2024-01-26

**Authors:** Emily L. Vogt, Shani Chibber, Charley Jiang, Rieham Owda, Martina T. Caldwell, Lisa H. Harris, Erica E. Marsh

**Affiliations:** 1 University of Michigan Medical School, Ann Arbor; 2 Division of Reproductive Endocrinology and Infertility, Department of Obstetrics and Gynecology, University of Michigan, Ann Arbor; 3 Department of Obstetrics and Gynecology, University of Texas Southwestern, Dallas; 4 Complex Family Planning Section, Division of Gynecology, Department of Obstetrics and Gynecology, University of Michigan, Ann Arbor; 5 Department of Emergency Medicine, Henry Ford Hospital, Detroit, Michigan; 6 Institute for Healthcare Policy and Innovation, University of Michigan, Ann Arbor; 7 Department of Women’s and Gender Studies, College of Literature, Sciences, and the Arts, University of Michigan, Ann Arbor; 8 Michigan Institute of Clinical and Health Research, University of Michigan, Ann Arbor

## Abstract

This cross-sectional study of female emergency contraception users examines emergency contraception–related emergency department use disparities and associations with policy changes.

## Introduction

Emergency contraception (EC) use increased after US Food and Drug Administration (FDA) approval for over-the-counter (OTC) use for adults on August 24, 2006, and minors in 2013 and the Patient Protection and Affordable Care Act mandate of EC insurance coverage in 2012.^1^ Emergency departments (EDs) are important sites for obtaining EC given their 24-hour access and high-acuity care. No study has examined EC-related ED utilization trends despite their utility as a barometer of OTC EC access. We report EC-related ED utilization disparities and associations with EC policy changes.

## Methods

This cross-sectional study analyzed EC-related ED utilization data from 2006 to 2020 from the Nationwide Emergency Department Sample, a database of 2 006 582 771 weighted US ED visits managed by the Healthcare Cost and Utilization Project of the Agency for Healthcare Research and Quality. Females aged 15 to 44 years with an *ICD-9* or *ICD-10* code of EC were included. Primary outcomes were annual EC-related ED visits and hospital charges with age, income quartile by zip code, hospital geographic region, payment method, and race and ethnicity assessed (eMethods in Supplement 1). We included race and ethnicity data, which were only available from 2019 to 2020, to assess pertinent disparities in EC ED utilization. We also assessed annual ED visits for any non-EC diagnosis to compare trends in overall vs EC-specific ED utilization. This study was exempted by the University of Michigan Institutional Review Board, with informed consent waived for the use of deidentified data. We followed the STROBE reporting guideline. We used SAS, version 9.4 (SAS Institute Inc) and Joinpoint, version 4.7.0.0 (National Cancer Institute) for analysis. We performed a χ^2^ test, 2-tailed *t* test, and 2020 US dollar inflation-adjusted analysis of hospital charge data, with 2-sided *P* < .05 defined as significant. Missing data were imputed to calculate total charges with age, region, income, and EC-related diagnosis covariates.

## Results

In total, 47 858 EC-related ED encounters occurred from 2006 to 2020. During this period, EC-related ED encounters decreased by 96%, from 17 019 to 659 (*P* < .001) (Figure), and total EC-related hospital charges decreased by 95% ($7.2 million), from $7.61 million to $385 946 (*P* < .001). The most notable decrease was from 2006 to 2007 and for a primary EC diagnosis. Younger, low-income, Black, Hispanic, and Medicaid-insured females were overrepresented in EC-related vs non-EC ED visits (Table). Northeast hospitals comprised 43.9% to 58.6% of EC-related ED visits despite comprising only 17.1% to 19.1% of non-EC ED visits. Southern hospitals comprised 4.5% to 17.4% of EC-related visits despite consistently averaging more than 40% of non-EC ED visits (Table). Individuals seeking care for sexual assault increased from 0.48% of EC-related ED visits in 2006 to 7.68% in 2020.

**Figure.**
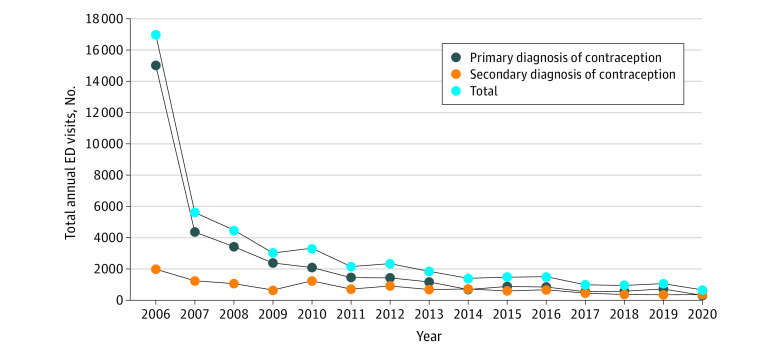
Total Annual Emergency Department (ED) Visits for Emergency Contraception Among Females Aged 15 to 44 Years From 2006 to 2020

**Table.** Total Annual ED Visits With Any Diagnosis of EC Compared With Total Annual Non-EC ED Visits Among Females Aged 15 to 44 Years From 2006 to 2020

Characteristic	No. (%) of EC ED visits									
	2006	2007	2008	2010	2012	2014	2016	2018	2019	2020
**EC ED visits**										
Total No.	17 019	5616	4491	3329	2345	1403	1509	941	1063	659
Age group, y										
15-19	5778 (34.0)	1722 (30.7)	1456 (32.4)	1041 (31.3)	601 (25.6)	433 (30.8)	415 (27.5)	294 (31.3)	296 (27.8)	186 (28.3)
20-24	6599 (38.8)	2252 (40.1)	1633 (36.3)	1205 (36.2)	775 (33.1)	359 (25.6)	479 (31.8)	250 (26.6)	256 (24.1)	158 (24.0)
25-29	2820 (16.6)	974 (17.3)	791 (17.6)	602 (18.1)	575 (24.5)	343 (24.4)	333 (22.1)	172 (18.3)	207 (19.4)	140 (21.2)
30-34	1074 (6.3)	381 (6.8)	412 (9.2)	255 (7.7)	262 (11.2)	179 (12.7)	163 (10.8)	147 (15.7)	164 (15.4)	106 (16.0)
35-39	512 (3.0)	193 (3.4)	149 (3.3)	153 (4.6)	101 (4.3)	54 (3.9)	86 (5.7)	45 (4.8)	100 (9.4)	49 (7.4)
40-44	236 (1.4)	93 (1.7)	50 (1.1)	72 (2.2)	30 (1.3)	36 (2.6)	34 (2.2)	32 (3.4)	41 (3.8)	21 (3.1)
Income quartile										
Lowest	3353 (19.7)	1867 (33.2)	1309 (29.1)	1375 (41.3)	790 (33.7)	483 (34.4)	568 (37.7)	446 (47.4)	339 (31.9)	219 (33.2)
Second	4078 (24.0)	1177 (21.0)	1350 (30.1)	873 (26.2)	536 (22.9)	406 (29.0)	349 (23.1)	221 (23.5)	293 (27.6)	124 (18.8)
Third	4339 (25.5)	1327 (23.6)	814 (18.1)	522 (15.7)	528 (22.5)	265 (18.9)	323 (21.4)	149 (15.8)	229 (21.5)	135 (20.4)
Highest	4776 (28.1)	1083 (19.3)	908 (20.2)	455 (13.7)	418 (17.8)	222 (15.8)	207 (13.7)	110 (11.7)	182 (17.1)	87 (13.2)
Payer										
Medicaid	2928 (17.2)	1399 (24.9)	1109 (24.7)	915 (27.5)	1206 (51.4)	705 (50.3)	838 (55.6)	557 (59.2)	694 (65.3)	366 (55.5)
Private	6989 (41.1)	2640 (47.0)	1657 (36.9)	1114 (33.5)	386 (16.5)	238 (16.9)	349 (23.1)	108 (11.5)	173 (16.3)	152 (23.0)
Medicare	135 (0.8)	50 (0.9)	79 (1.8)	87 (2.6)	48 (2.0)	48 (3.4)	52 (3.5)	18 (1.9)	41 (3.9)	18 (2.7)
Self-pay	4637 (27.2)	1265 (22.5)	1187 (26.4)	842 (25.3)	366 (15.6)	174 (12.4)	251 (16.6)	230 (24.5)	131 (12.3)	74 (11.2)
No charge	1473 (8.7)	81 (1.4)	47 (1.0)	4 (0.1)	4 (0.2)	4 (0.3)	4 (0.3)	0 (0.0)	0 (0.0)	0 (0.0)
Other	603 (3.5)	153 (2.7)	377 (8.4)	352 (10.6)	327 (14.0)	234 (16.7)	14 (0.9)	22 (2.3)	24 (2.3)	50 (7.6)
Hospital region										
Northeast	9868 (58.0)	3290 (58.6)	2569 (57.2)	1813 (54.5)	1346 (57.4)	741 (52.8)	763 (50.6)	484 (51.5)	604 (56.8)	290 (43.9)
South	2542 (14.9)	635 (11.3)	694 (15.4)	274 (8.2)	105 (4.5)	95 (6.7)	76 (5.1)	78 (8.3)	100 (9.4)	115 (17.4)
Midwest	2237 (13.1)	836 (14.9)	645 (14.4)	546 (16.4)	438 (18.7)	319 (22.7)	303 (20.1)	114 (12.1)	123 (11.6)	128 (19.4)
West	2371 (13.9)	854 (15.2)	583 (13.0)	696 (20.9)	455 (19.4)	249 (17.7)	366 (24.3)	265 (28.2)	236 (22.2)	127 (19.3)
Race and ethnicity										
Asian or Pacific Islander	NA	NA	NA	NA	NA	NA	NA	NA	27 (1.6)	11 (0.6)
Black	NA	NA	NA	NA	NA	NA	NA	NA	364 (21.6)	266 (15.8)
Hispanic	NA	NA	NA	NA	NA	NA	NA	NA	273 (16.2)	141 (8.4)
Native American	NA	NA	NA	NA	NA	NA	NA	NA	34 (2.0)	36 (2.1)
White	NA	NA	NA	NA	NA	NA	NA	NA	253 (15.0)	162 (9.6)
Other	NA	NA	NA	NA	NA	NA	NA	NA	92 (5.5)	27 (1.6)
**Non-EC ED Visits**										
Total	30 163 819	30 860 401	31 749 810	33 249 231	33 794 672	34 734 293	35 474 680	33 669 056	33 460 351	28 840 073
Age group, y										
15-19	4 756 348 (15.8)	4 840 044 (15.7)	4 928 087 (15.5)	4 841 302 (14.6)	4 709 069 (13.9)	4 607 453 (13.3)	4 918 617 (13.9)	4 751 216 (14.1)	4 514 696 (13.5)	3 681 856 (12.8)
20-24	6 291 324 (20.9)	6 429 684 (20.8)	6 666 266 (21.0)	7 171 726 (21.6)	7 203 487 (21.3)	7 196 254 (20.7)	6 955 800 (19.6)	6 395 561 (19.0)	6 268 122 (18.7)	5 378 206 (18.6)
25-29	5 551 181 (18.4)	5 856 032 (19.0)	6 109 108 (19.2)	6 496 426 (19.5)	6 602 611 (19.5)	6 934 624 (20.0)	7 108 625 (20.0)	6 722 651 (20.0)	6 618 605 (19.8)	5 601 605 (19.4)
30-34	4 558 090 (15.1)	4 699 907 (15.2)	4 898 517 (15.4)	5 433 678 (16.3)	5 821 859 (17.2)	6 155 197 (17.7)	6 273 434 (17.7)	5 955 614 (17.7)	6 059 033 (18.1)	5 345 630 (18.5)
35-39	4 478 866 (14.8)	4 548 809 (14.7)	4 651 104 (14.6)	4 746 842 (14.3)	4 777 734 (14.1)	5 090 178 (14.7)	5 496 749 (15.5)	5 353 381 (15.9)	5 386 799 (16.1)	4 724 128 (16.4)
40-44	4 528 011 (15.0)	4 485 925 (14.5)	4 496 728 (14.2)	4 559 256 (13.7)	4 679 912 (13.8)	4 750 587 (13.7)	4 721 455 (13.3)	4 490 633 (13.3)	4 613 096 (13.8)	4 108 648 (14.2)
Income quartile										
Lowest	9 767 889 (32.4)	10 520 600 (34.1)	10 509 664 (33.1)	11 665 132 (35.1)	11 910 271 (35.2)	12 848 822 (37.0)	13 267 656 (37.4)	12 921 480 (38.4)	12 439 976 (37.2)	10 654 135 (36.9)
Second	8 204 926 (27.2)	8 383 184 (27.2)	9 476 084 (29.8)	9 303 828 (28.0)	8 955 209 (26.5)	10 012 982 (28.8)	9 752 807 (27.5)	9 355 222 (27.8)	8 749 789 (26.1)	7 914 030 (27.4)
Third	6 704 086 (22.2)	6 747 213 (21.9)	6 443 822 (20.3)	6 922 773 (20.8)	7 536 316 (22.3)	6 760 723 (19.5)	7 041 931 (19.9)	6 411 367 (19.0)	7 042 658 (21.0)	5 676 676 (19.7)
Highest	4 865 716 (16.1)	4 516 612 (14.6)	4 554 195 (14.3)	4 688 074 (14.1)	4 833 266 (14.3)	4 497 758 (12.9)	4 912 667 (13.8)	4 541 220 (13.5)	4 788 115 (14.3)	4 169 154 (14.5)
Payer										
Medicaid	9 046 514 (30.0)	8 830 817 (28.6)	9 498 463 (29.9)	11 486 248 (34.5)	12 720 089 (37.6)	14 842 769 (42.7)	15 074 329 (42.5)	14 488 983 (43.0)	13 706 927 (41.0)	11 931 059 (41.4)
Private	11 522 493 (38.2)	11 796 833 (38.2)	11 973 780 (37.7)	11 102 849 (33.4)	10 177 162 (30.1)	10 591 869 (30.5)	11 637 884 (32.8)	11 012 438 (32.7)	11 248 588 (33.6)	10 044 987 (34.8)
Medicare	1 139 373 (3.8)	1 196 092 (3.9)	1 268 620 (4.0)	1 388 079 (4.2)	1 590 884 (4.7)	1 620 439 (4.7)	1 609 347 (4.5)	1 357 798 (4.0)	1 397 012 (4.2)	1 140 877 (4.0)
Self-pay	6 435 993 (21.3)	7 178 675 (23.3)	6 959 087 (21.9)	7 319 805 (22.0)	7 248 483 (21.4)	5 853 036 (16.9)	5 337 260 (15.0)	5 349 905 (15.9)	5 538 317 (16.6)	4 246 915 (14.7)
No charge	348 949 (1.2)	280 134 (0.9)	297 802 (0.9)	261 840 (0.8)	212 190 (0.6)	232 291 (0.7)	181 056 (0.5)	140 555 (0.4)	199 378 (0.6)	186 679 (0.6)
Other	1 549 003 (5.1)	1 409 548 (4.6)	1 563 672 (4.9)	1 543 587 (4.6)	1 786 586 (5.3)	1 514 965 (4.4)	1 574 599 (4.4)	1 288 577 (3.8)	1 310 323 (3.9)	1 235 397 (4.3)
Hospital region										
Northeast	5 704 610 (18.9)	5 864 417 (19.0)	6 068 541 (19.1)	6 110 390 (18.4)	6 188 372 (18.3)	6 160 816 (17.7)	6 295 084 (17.7)	5 927 723 (17.6)	5 829 979 (17.4)	4 942 714 (17.1)
South	12 368 393 (41.0)	12 660 327 (41.0)	13 023 502 (41.0)	13 669 244 (41.1)	14 125 083 (41.8)	14 681 573 (42.3)	14 726 510 (41.5)	14 192 806 (42.2)	14 052 034 (42.0)	12 287 991 (42.6)
Midwest	7 023 309 (23.3)	7 454 562 (24.2)	7 637 822 (24.1)	8 101 006 (24.4)	7 903 343 (23.4)	8 014 762 (23.1)	8 269 693 (23.3)	7 529 387 (22.4)	7 571 854 (22.6)	6 377 999 (22.1)
West	5 067 507 (16.8)	4 881 095 (15.8)	5 019 945 (15.8)	5 368 591 (16.1)	5 577 874 (16.5)	5 877 141 (16.9)	6 183 393 (17.4)	6 019 140 (17.9)	6 006 484 (18.0)	5 231 368 (18.1)
Race and ethnicity										
Asian and Pacific Islander	NA	NA	NA	NA	NA	NA	NA	NA	676 202 (1.1)	616 224 (1.0)
Black	NA	NA	NA	NA	NA	NA	NA	NA	8 553 026 (14.0)	7 210 847 (11.8)
Hispanic	NA	NA	NA	NA	NA	NA	NA	NA	5 823 904 (9.5)	5 083 289 (8.3)
Native American	NA	NA	NA	NA	NA	NA	NA	NA	203 612 (0.3)	205 122 (0.3)
White	NA	NA	NA	NA	NA	NA	NA	NA	16 341 853 (26.8)	14 008 364 (23.0)
Other^a^	NA	NA	NA	NA	NA	NA	NA	NA	1 218 725 (2.0)	1 070 615 (1.8)

Abbreviations: EC, emergency contraception; ED, emergency department; NA, not available.

^a^ Other includes any racial or ethnic group not listed or multiracial.

## Discussion

Our study demonstrates a large decrease in EC-related ED visits and a $7.2 million total annual decrease in hospital charges coinciding with FDA approval (Figure). Although this decrease may have started before 2006, the magnitude of this 2006 to 2007 decline with FDA OTC approval in late August 2006 suggests an association. Our finding that younger, low-income, Medicaid-insured, Black, and Hispanic females are overrepresented in EC-related ED visits aligns with a previous outpatient study^2^ and suggests ongoing barriers to OTC EC access and/or increased ED utilization for other reasons, including sexual assault.^3^ The steep decrease in privately insured EC ED visits affirms possible income-related disparities.^4^ Southern hospitals were disproportionately underrepresented in EC ED visits, aligning with previously defined geographic disparities in OTC EC access.^4,5^

Study strengths include sample size, national scope, and analysis of insurance and income-related variables and race. Limitations include lack of data before 2006, limited race and ethnicity data, and reliance on *ICD* codes to approximate services.

Increasingly restrictive abortion access will likely drive EC demand to prevent unintended pregnancy. Moreover, disparities in EC-related ED utilization highlight complexities in OTC EC for certain populations. Future policies should reduce barriers to make EC safe and affordable to all.

## Notes

Supplement 1. **eMethods.** Supplementary Methods

Supplement 2.
Data Sharing Statement
