# Supplementary material for: Trends in Encounters for Emergency Contraception in US Emergency Departments, 2006-2020
Source: JAMA Netw Open. 2024 Jan 26;7(1):e2353672. doi: 10.1001/jamanetworkopen.2023.53672 (PMC10818211; doi:10.1001/jamanetworkopen.2023.53672)
Supplement: Supplement 1. — eMethods. Supplementary Methods [file jamanetwopen-e2353672-s001.pdf]

Supplementary Online Content

Vogt EL, Chibber S, Jiang C, et al. Trends in encounters for emergency conception in US emergency departments, 2006-2020. *JAMA Netw Open*. 2024;7(1):e2353672. doi:10.1001/jamanetworkopen.2023.53672

eMethods. Supplementary Methods

This supplementary material has been provided by the authors to give readers additional information about their work.

© 2024 Vogt EL et al. *JAMA Network Open.*

eMethods. Supplementary Methods

The Nationwide Emergency Department Sample (NEDS) includes over 28 million US emergency department (ED) visits from 995 hospitals across 40 states and the District of Columbia, yielding a 20% stratified sample of hospital owned EDs. State participation is voluntary and varies annually, which may impact some of the trends in year-over-year data. NEDS currently has available data from 2006 through 2020.

The specific ICD-9 and ICD-10 code used for sample selection was "encounter for emergency contraception counseling and prescription" for any visit for any female aged 15-44 from January 1, 2006 through December 31, 2020. Of note, NEDS reports ICD-9 codes from 2006 through September 30, 2015 and ICD-10 codes from October 1, 2015 through 2020 (https://hcup-us.ahrq.gov/db/nation/neds/nedsdde.jsp).

Racial and ethnicity categories included Asian or Pacific Island, Black, Hispanic, Native American, White, or Other as defined by the Healthcare Cost and Utilization Project (HCUP) of the Agency for Healthcare Research and Quality. These categories were reported by participating hospital sites at the state level. The exact definition of these categories, including the Other category, varied on a state-by-state basis with more details included on the HCUP website (https://hcup-us.ahrq.gov/db/vars/race/nedsnote.jsp). HCUP only began collecting these data from hospital partners in 2019, which is why we have only included race and ethnicity data for 2019-2020.

© 2024 Vogt EL et al. *JAMA Network Open.*
