# Supplementary material for: Trends in Encounters for Emergency Contraception in US Emergency Departments, 2006-2020
Source: JAMA Netw Open. 2024 Jan 26;7(1):e2353672. doi: 10.1001/jamanetworkopen.2023.53672 (PMC10818211; doi:10.1001/jamanetworkopen.2023.53672)
Supplement: Supplement 2. — Data Sharing Statement [file jamanetwopen-e2353672-s002.pdf]

Data Sharing Statement

Vogt. Trends in Encounters for Emergency Contraception in US Emergency Departments, 2006-2020. *JAMA Netw Open*. Published January 26, 2024.
doi:10.1001/jamanetworkopen.2023.53672

Data
Data available: No

Additional Information
Explanation for why data not available: These data are available to the public from HCUP for purchase but under their contract, we cannot share their data.
